# Identification of a 15-pseudogene based prognostic signature for predicting survival and antitumor immune response in breast cancer

**DOI:** 10.18632/aging.103735

**Published:** 2020-12-16

**Authors:** Liqiang Tan, Xiaofang He, Guoping Shen

**Affiliations:** 1 Department of Medical Bioinformatics, Zhongshan School of Medicine, Sun Yat-sen University, Guangzhou 510080, China; 2 Center for Stem Cell Biology and Tissue Engineering, Key Laboratory for Stem Cells and Tissue Engineering, Ministry of Education, Sun Yat-sen University, Guangzhou 510080, China; 3 Department of Radiation Oncology, The First Affiliated Hospital of Sun Yat-sen University, Guangzhou 510080, China; 4 Department of Cancer Biology, Dana-Farber Cancer Institute, Boston, MA 02115, USA

**Keywords:** pseudogene, breast cancer, survival, risk score, antitumor immune response

## Abstract

Pseudogenes are noncoding RNAs that have been revealed to play critical roles in oncogenesis and tumor progression. However, their functional roles have not been comprehensively clarified in breast cancer. Here, we systematically analyzed the RNA sequencing data of 13931 pseudogenes in 775 breast cancer patients from The Cancer Genome Atlas dataset, and ultimately identified 15 prognostic pseudogenes by univariate Cox proportional hazard regression. A risk score model was constructed based on the prognostic pseudogenes via LASSO analysis and dichotomized patients into low- and high-risk subgroups. Patients in the high-risk group had a significantly shorter overall survival than those in the low-risk group. The prognostic value of these 15 pseudogenes and the risk score model were further validated in the European Genome-Phenome Archive dataset. Furthermore, we performed consensus clustering of the 15 prognostic pseudogenes and found that their expression pattern was significantly associated with tumor malignancy and host antitumor immune response, in terms of infiltrating immune cell compositions, antigen presenting genes expression, cytolytic activity and T-cell exhausted markers. This study indicated that these 15 prognostic pseudogenes were significantly correlated with tumor malignancy and host antitumor immune response in breast cancer, and might serve as potential targets for immunotherapy.

## INTRODUCTION

Breast cancer is the most common malignant tumor and the second leading cause of death for females globally. Nowadays, the robust predictive factors for prognosis of breast cancer patients are two clinical features—tumor size and lymph node status at the time of detection [1]. Carcinomas with large tumor size or lymph node metastasis are usually associated with poor survival outcomes. However, breast cancers are well known as highly heterogenous neoplasms and driven by complex signaling pathways [2], which in part accounts for the fact that different therapeutic responses and then different survival outcomes can be observed even in patients diagnosed with the same breast cancer molecular subtype [3] and TNM stage. Therefore, looking for additional promising prognostic biomarkers, especially in the intrinsic molecular level [4, 5], is imperative so as to identify high-risk subgroups and make precise therapeutic strategies.

Nowadays, the standard of care for primary breast cancer is surgery, followed by chemotherapy, endocrine therapy, radiotherapy and targeted therapy on the basis of molecular subtypes and TNM stage. While in recent years, immunotherapy is emerging as a novel treatment modality due to the promising therapeutic effect of selective immune checkpoint inhibitors in combination with other strategies [6], especially monoclonal antibodies against programmed death 1 (PD-1), programmed death-ligand 1 (PD-L1) and cytotoxic T lymphocyte-associated protein-4 axes (CTLA-4) [7]. As of September, 2018, the number of registered trials that are open to breast cancer patients, which assess novel approaches by harnessing the immune system, has reached up to 285 [8–10]. At the current stage, the expression level of PD-L1 in tumor tissue is a commonly-used predictive marker for immune response [11, 12]. However, the predictive results were not satisfactory, which indicates that immune modulation is a complicated process and requires much more functional predictors [13–15]. Therefore, it is essential to identify robust biological predictive markers of immune response when conducting clinical trials of immunotherapy.

Pseudogenes are non-coding homologs of protein-coding genes, which are often caused by accumulation of multiple mutations within genes, and their products are nonfunctional [16]. Pseudogenes were once labeled as “genetic fossils” because of lack of protein-coding ability or cellular gene expression. However, due to the development of high-throughput sequencing technologies, pseudogenes have been revealed to participant in various biological functions by regulating their parental transcripts, acting as competitive endogenous RNAs (ceRNA) [17–19]. What’s more, the significance of pseudogenes in gene regulation has also been highlighted in tumorigenesis and tumor progression recently [20, 21], which was largely attributed to the finding that PTEN pseudogene 1 could upregulate his parental gene PTEN, a well-known tumor suppressor, via ceRNA mechanism and thus played a pivotal role in tumorigenesis in breast cancer [22]. However, until now, it has not been comprehensively clarified the prognostic effect of pseudogenes in patients with breast cancer, and their potential roles in host antitumor immune response remain largely unexplored.

Based on the concerns mentioned above, we systematically analyzed the RNA sequencing data of pseudogenes in 775 patients with breast cancer from The Cancer Genome Atlas (TCGA) dataset and eventually identified 15 prognostic indicators, which were further validated using the European Genome-Phenome Archive (EGA) dataset. A risk score model was constructed based on the prognostic pseudogenes, and their expression pattern was functionally annotated by Gene Ontology (GO), Kyoto Encyclopedia of Genes and Genomes (KEGG) analyses and Gene Set Enrichment Analysis (GSEA). Besides, we also investigated the association between the prognostic pseudogenes and the host antitumor immune response, in terms of tumor-infiltrating immune cell compositions, antigen presenting genes expression, immunomodulator genes expression and cytolytic activity, so as to provide potential predictive markers to immunotherapy in breast cancer.

## RESULTS

### Identification of 15 prognostic pseudogenes

**Figure 1:**
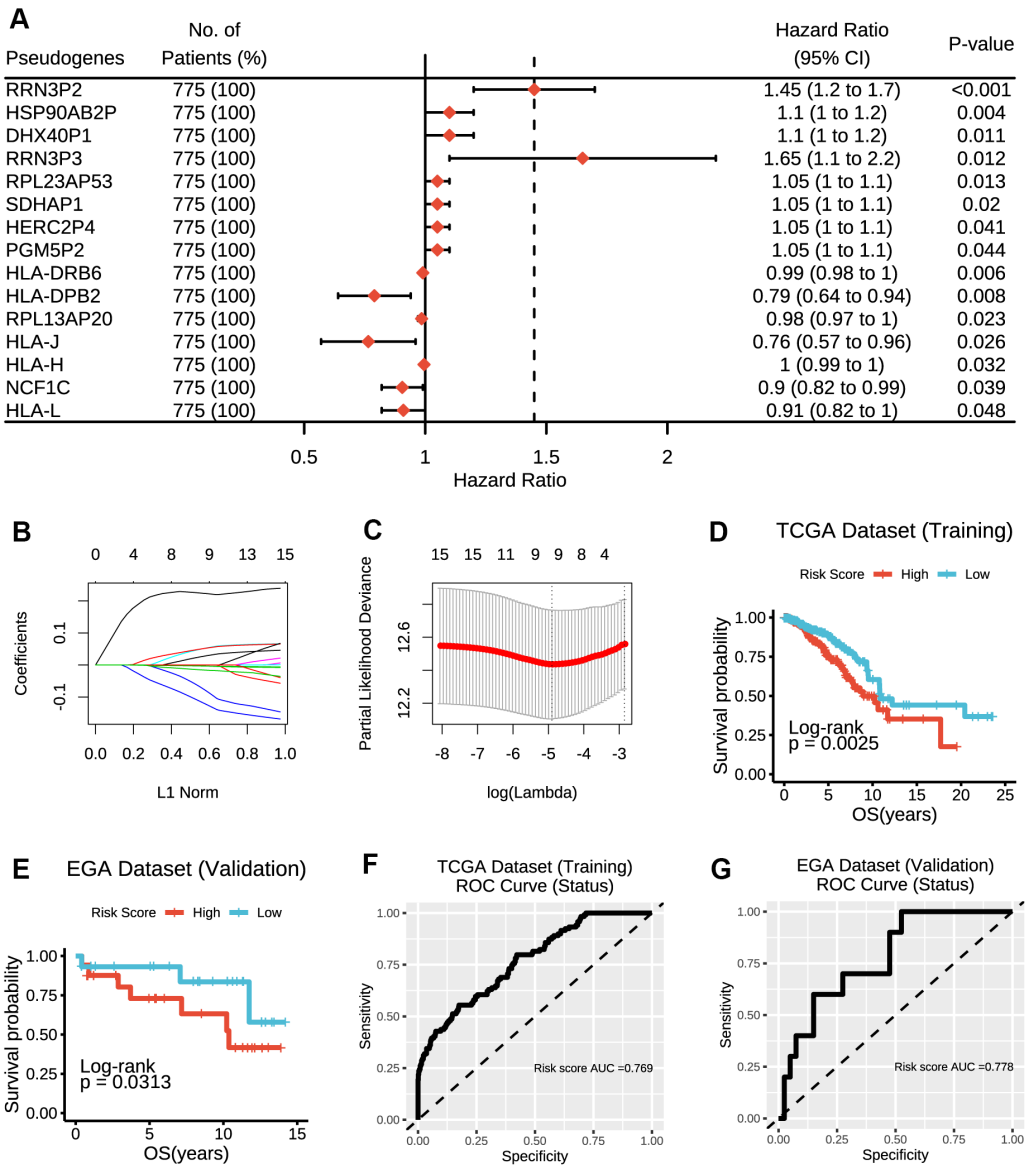
**Construction of the risk score model based on prognostic pseudogenes.** (**A**) The hazard ratios (HR), 95% confidence intervals (CI) calculated by univariate Cox proportional hazard regression of 15 prognostic pseudogenes using TCGA data. (**B**) LASSO coefficient profiles of 15 prognostic pseudogenes. (**C**) Ten-time cross-validation for tuning parameter selection in the LASSO model of 15 prognostic pseudogenes. (**D**) The breast cancer patients from TCGA dataset in high-risk group displayed significantly shorter overall survival than those in low-risk group (p = 0.0025). (**E**) The breast cancer patients from EGA dataset in high-risk group displayed significantly shorter overall survival than those in low-risk group (p = 0.0313). (**F**) The ROC curve and AUC for the risk score model in TCGA dataset. (**G**) The ROC curve and AUC for the risk score model in EGA dataset.

Altogether, a whole list of 13931 pseudogenes were obtained from Vega databases and psiCube databases, of which 308 pseudogenes were available in TCGA datasets and thus included in the subsequent analyses. As results of univariate Cox proportional hazard regression indicated, a total of 15 pseudogenes were ultimately identified to be significantly associated with survival outcomes in TCGA dataset (Figure 1A), which was further verified in EGA dataset (Supplementary Figure 1).

### Construction of a risk signature based on the prognostic pseudogenes

To improve the predictive effect of pseudogenes in the clinical outcomes of breast cancer, we applied the least absolute shrinkage and selection operator (LASSO) Cox regression algorithm to the 15 prognostic pseudogenes and constructed a risk signature based on the minimum criteria using TCGA data as the training set (Figure 1B, 1C) and EGA data as the validation set. The coefficients of the 15 pseudogenes were listed in Supplementary Table 1. The risk score was calculated according to survival risk score model formula. Then, the breast cancer patients were dichotomized into low or high-risk groups according to the median risk score. Results indicated that patients in high-risk group displayed significantly shorter overall survival than those in low-risk group (TCGA dataset, median overall survival 8.94 years vs. 10.85 years, log-rank test, p = 0.0025, Figure 1D; EGA dataset, median overall survival 10.79 years vs. 12.77 years, log-rank test, p = 0.0313, Figure 1E). The ROC curves showed that the risk score was good to predict survival rates with an AUC value of 0.769 in the training set (Figure 1F) and 0.778 in the validation set (Figure 1G). In addition, RRN3P2 and HLA-DRB6 were found to have significant associations with overall survivals. Patients with high expression of RRN3P2 had significantly shorter survival than those with low expression (median overall survival 8.94 years vs. 11.69 years, log-rank test, p = 0.0088, Supplementary Figure 2A), indicating that high expression of RRN3P2 might correlate with high malignancy. On the contrary, patients with high expression of HLA-DRB6 had significantly longer survival than those with low expression (median overall survival 20.42 years vs. 10.24 years, log-rank test, p = 0.014, Supplementary Figure 2B). Besides, RPL23AP53, HLA-DRB6, RPL13AP20, NCF1C and HLA-L were found to have significant associations with overall survivals in EGA dataset (Supplementary Figure 3A–3E).

### Expressions of prognostic pseudogenes significantly associated with different clinicopathological features and survival outcomes

**Figure 2:**
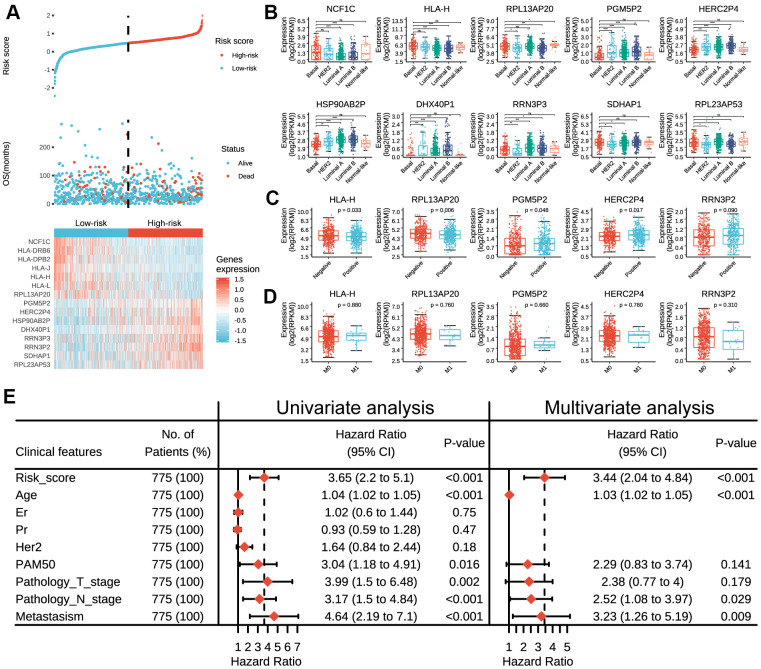
**Expressions of prognostic pseudogenes in breast cancer by different clinicopathological features in TCGA dataset.** (**A**) The distribution of risk score, vital status and the expression pattern of 15 prognostic pseudogenes in 775 breast cancer patients. The risk scores are arranged in ascending order from left to right. (**B**) Expression levels of NCF1C, HLA-DRB6, HLA-DPB2, HLA-J, HLA-H, HLA-L, RPL13AP20, PGM5P2, HERC2P4, HSP90AB2P, DHX40P1, RRN3P3, RRN3P2, SDHAP1 and RPL23AP53 across different breast cancer subtypes. (**C**) Expression levels of HLA-H, RPL13AP20, PGM5P2, HERC2P4 and RRN3P2 in patients with or without lymph node metastasis. (**D**) Expression levels of HLA-H, RPL13AP20, PGM5P2, HERC2P4 and RRN3P23 in patients with or without distant metastasis. (**E**) Univariate and multivariate Cox regression analyses of the association between clinicopathological factors (including the risk score) and overall survival of breast cancer patients. ns denotes no significance, *** denotes P < 0.001 and **** denotes P < 0.0001.

The distribution of the risk scores, overall survival, and corresponding pseudogene expression profiles in TCGA dataset were demonstrated in Figure 2A. Heatmap indicated that NCF1C, HLA-DRB6, HLA-DPB2, HLA-J, HLA-H, HLA-L and RPL13AP20 displayed high expressions in the low-risk group, and thus were categorized as tumor-suppressor pseudogenes in the current study. On the other hand, the remaining pseudogenes (PGM5P2, HERC2P4, HSP90AB2P, DHX40P1, RRN3P3, RRN3P2, SDHAP1 and RPL23AP53) displayed high expressions in the high-risk group and thus were categorized as oncogene pseudogenes in the current study. Besides, we also found that the risk score and prognostic pseudogenes were closely related to different clinicopathological features of breast cancer patients. The low-risk group was significantly associated with ER status (p = 8e-08), PR status (p = 6e-04), more basal-like molecular subtype (p = 4e-06) and lower lymph node stage (p = 0.037) compared with high-risk group (Supplementary Table 2). Basal-like subtype had significantly higher expressions of NCF1C, HLA-H, RPL13AP20 RRN3P3 and SDHAP1, but lower expressions of PGM5P2, HSP90AB2P and DHX40P1 than other subtypes (Figure 2B). In addition, patients with lymph node metastasis had significantly higher expressions of PGM5P2, HERC2P4 and RRN3P2 but lower expressions of HLA-H and RPL13AP20 than those without lymph node metastasis (Figure 2C). There were no significant differences in the expressions of the 15 prognostic pseudogenes between patients with or without distant metastasis (Figure 2D).

Furthermore, univariate Cox regression analysis demonstrated that risk score, age, PAM50, pathology T stage, pathology N stage and metastasis status were all correlated with the overall survival. When including these factors in the multivariate Cox regression, we found that risk score (p < 0.001), age (p < 0.001), pathology N stage (p = 0.029) and metastasis status (p = 0.009) remained significantly associated with the clinical outcome (Figure 2E), which indicated that the risk score derived from these 15 pseudogenes could independently predict prognosis in breast cancer patients.

### Consensus clustering of prognostic pseudogenes identified two clusters highly consistent with that of the risk score

**Figure 3:**
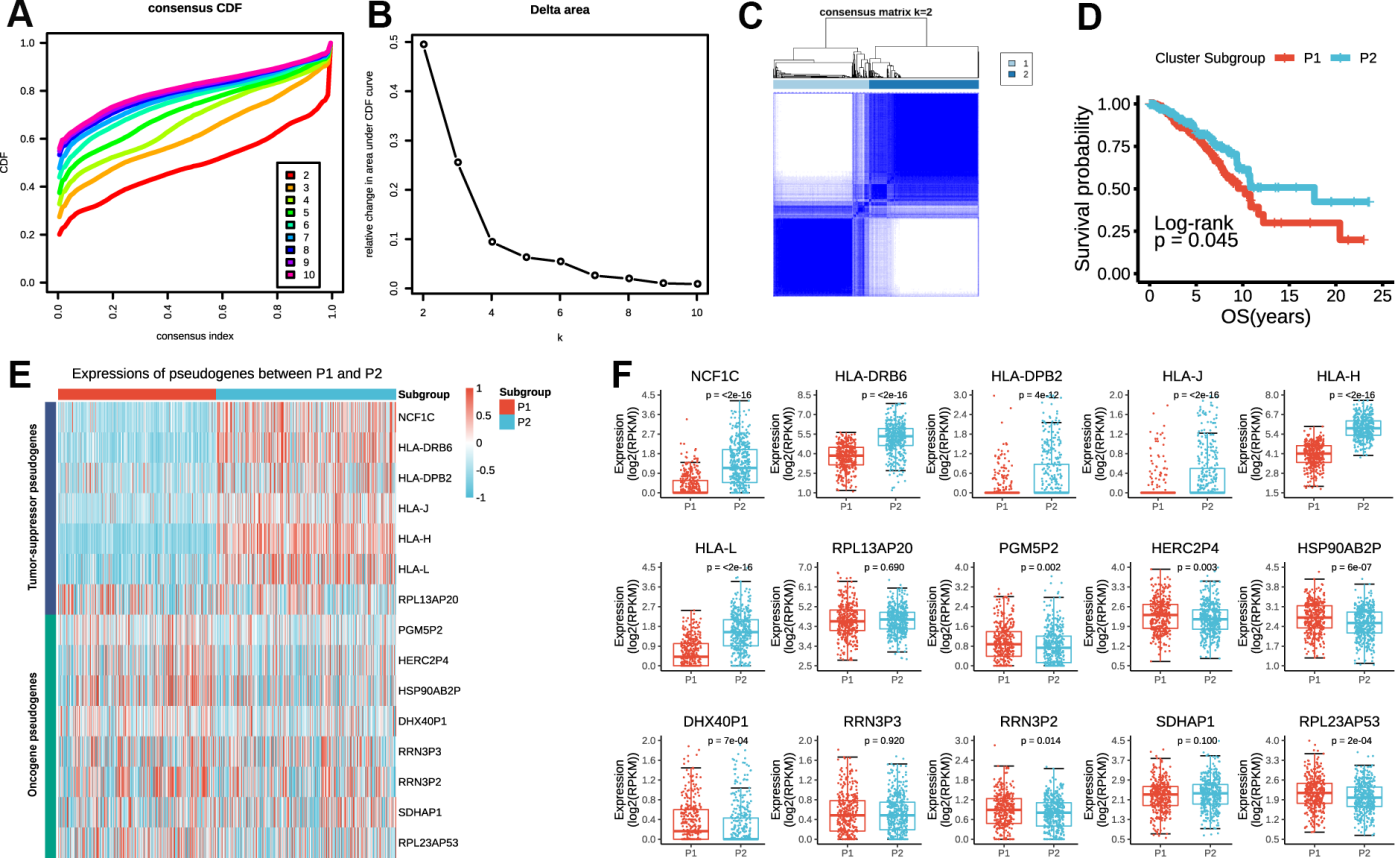
**P1/2 subgroups identified by consensus clustering of the 15 prognostic pseudogenes in TCGA dataset.** (**A**) Consensus clustering cumulative distribution function (CDF) for k = 2 to 10. (**B**) Relative change in area under CDF curve for k = 2 to 10. (**C**) Consensus clustering of 775 breast cancers with k = 2. (**D**) Kaplan-Meier overall survival (OS) curves for patients in P1 and P2 subgroups. (**E**) The expression pattern of prognostic pseudogenes between P1 and P2 subgroups. (**F**) Expression levels of NCF1C, HLA-DRB6, HLA-DPB2, HLA-J, HLA-H, HLA-L, RPL13AP20, PGM5P2, HERC2P4, HSP90AB2P, DHX40P1, RRN3P3, RRN3P2, SDHAP1 and RPL23AP53 between P1 and P2 subgroups.

Considering the large amounts of prognostic pseudogenes, we adopted dimensionality reduction analysis through consensus clustering of the 15 prognostic pseudogenes in the subsequent analysis. According to the expression similarity of pseudogenes, k = 2 seemed to be the optimal selection when clustering stability increased from k = 2 to 10 in the TCGA dataset (Figure 3A–3C). Therefore, we divided the 775 breast cancer patients into two subgroups by making 2 as the k value, namely, P1 (Patients subgroup 1) and P2 (Patients subgroup 2). Kaplan-Meier analysis revealed that patients in P2 subgroup had significantly longer overall survival than those in P1 subgroup (median overall survival 17.69 years vs. 10.24 years, log-rank test, p = 0.045, Figure 3D). The expression pattern of the 15 prognostic pseudogenes across P1 and P2 subgroups was displayed in Figure 3E. Results indicated that P1 subgroup had lower expressions of tumor-suppressor pseudogenes and higher expressions of oncogene pseudogenes, while P2 subgroup showed the opposite trends. What’s more, compared with P1, P2 subgroup had significantly higher expressions of 6 tumor-suppressor pseudogenes (NCF1C, p < 2e-16; HLA-DRB6, p < 2e-16; HLA-DPB2, p = 4e-12; HLA-J, p < 2e-16; HLA-H, p < 2e-16; HLA-L, p < 2e-16), and significantly lower expressions of 6 oncogene pseudogenes (PGM5P2, p = 0.002; HERC2P4, p = 0.003; HSP90AB2P, p = 6e-07; DHX40P1, p = 7e-04; RRN3P2, p = 0.014; RPL23AP53, p = 2e-04) (Figure 3F). In addition, we found that the P1 and P2 subgroup were also significantly associated with the clinicopathological features. P2 subgroup was significantly associated with ER status (p = 0.028), more basal-like molecular subtype (p = 0.002) and lower lymph node stage (p = 0.047) compared with P1 (Supplementary Table 3). These findings were highly consistent with those of the risk score mentioned above, which indicated that the expression pattern of prognostic pseudogenes was significantly associated with tumor survival.

### Expression pattern of prognostic pseudogenes was closely associated with malignancy of breast cancer

**Figure 4:**
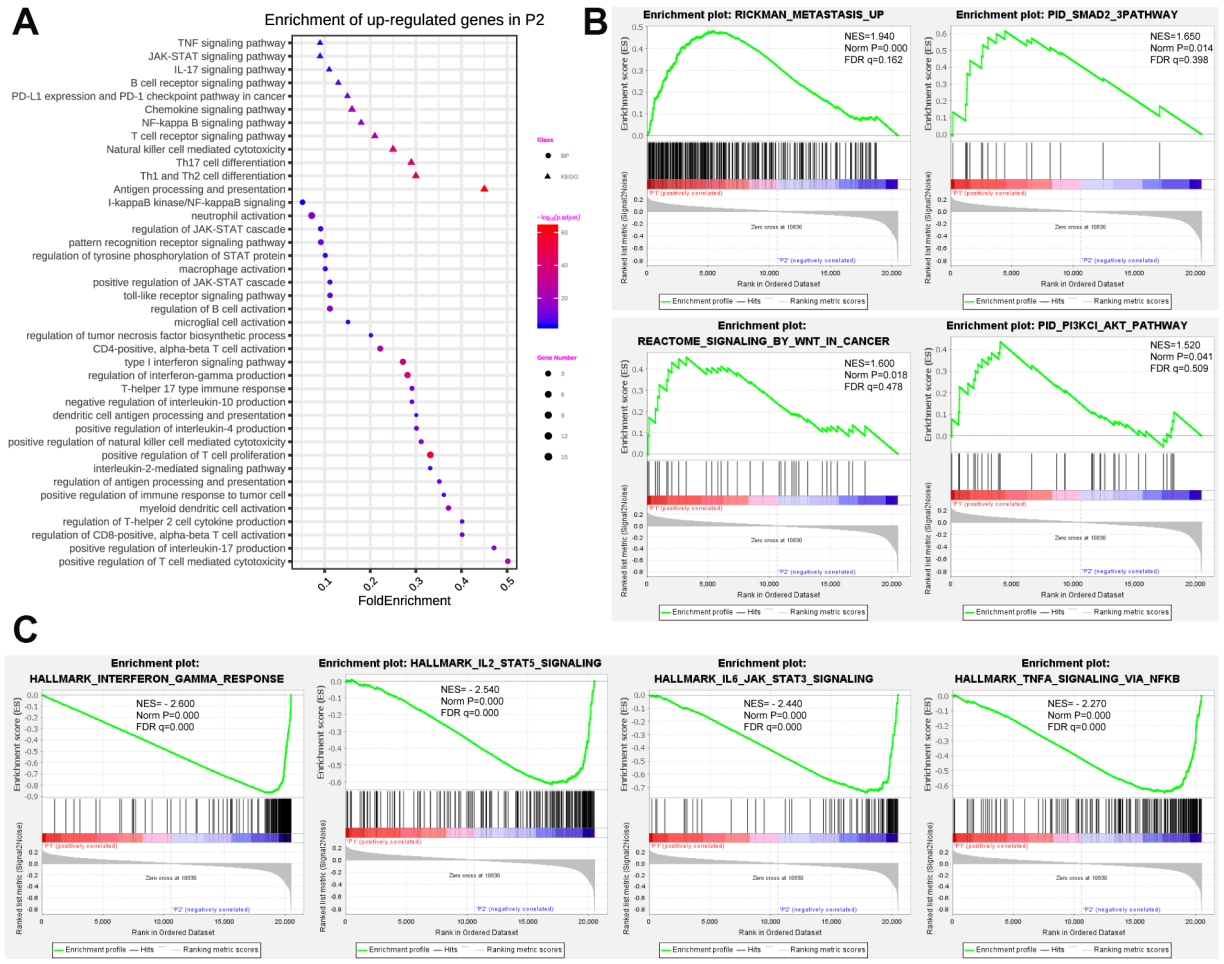
**Functional annotation of differentially expressed genes in P1/P2 subgroups.** (**A**) Functional annotation of up-regulated genes in P2 subgroup compared with P1 by using GO in terms of biological process (BP) and KEGG signaling pathway. (**B**) GSEA revealed that up-regulated genes in P1 subgroup were enriched for hallmarks of malignant tumors. (**C**) GSEA revealed that up-regulated genes in P2 subgroup were enriched for hallmarks of antitumor immune response.

To better illuminate the association between prognostic pseudogenes and malignancy of breast cancer, we identified the differentially expressed genes between P1 and P2 subgroups and annotated their functions using GO, KEGG pathway analysis and GSEA. GO pathway analyses revealed that upregulated genes in P2 were significantly enriched in tumor-related biological processes and pathways (Figure 4A), including regulation of JAK-STAT cascade, pattern recognition receptor signaling pathway and type I interferon signaling pathway. KEGG pathway analysis indicated that upregulated genes in P2 were enriched in TNF, JAK-STAT, IL-17, B cell receptor, Chemokine, NF-kappa B, T cell receptor signaling pathway and PD-L1expression and PD-1 checkpoint pathway in cancer (Figure 4A). Furthermore, “METASTASIS”, “SMAD”, “SIGNALING_BY_WNT_IN_CANCER” and “PI3KCI_AKT” were strikingly enriched in P1 subgroup indicating by GSEA (Figure 4B), while the hallmarks of “INTERFERON GAMMA RESPONSE”, “IL2 STAT5 SIGNALING”, “IL6 JAK STAT3 SIGNALING”, and “TNF SIGNALING” were remarkably enriched in P2 subgroup (Figure 4C). All these results partially clarified the mechanism underlying the prognostic effect of pseudogenes in breast cancer.

### Expression pattern of prognostic pseudogenes was significantly correlated with antitumor immune response

To investigate the correlation between the expression pattern of pseudogenes and antitumor immune response in breast cancer, we assessed the immune cell infiltration using CIBERSORT, and estimated the expressions of antigen presentation genes, cytolytic genes and immunomodulator genes in tumor tissues between P1 and P2 subgroups.

**Figure 5:**
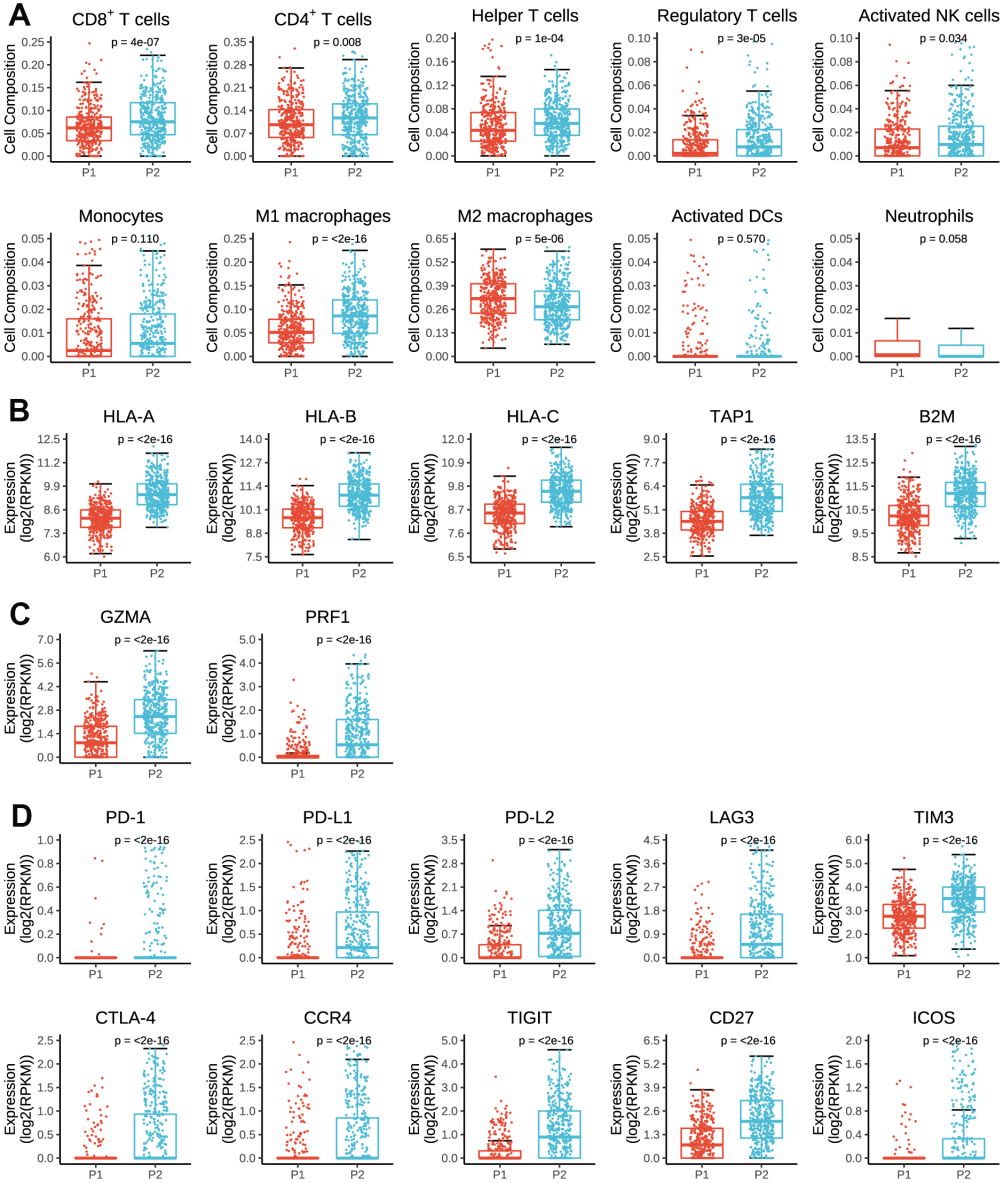
**Immune cell infiltration and expressions of antigen presenting genes, immune cytolysis genes and immunomodulator genes in tumor tissues by P1/P2 subgroups.** (**A**) Comparisons of cell composition fraction of CD8+ T cells, CD4+ T cells, helper T cells, regulatory T cells, activated natural killer (NK) cells, monocytes, M1 macrophages, M2 macrophages, activated dendritic cells (activated DCs) and neutrophils between P1 and P2 subgroups. (**B**) Expressions of HLA-A, HLA-B, HLA-C, TAP1 and B2M between P1 and P2 subgroups. (**C**) Expressions of GZMA and PRF1 between P1 and P2 subgroups. (**D**) Expressions of PD-1, PD-L1, PD-L2, LAG3, TIM3, CTLA-4, CCR4, TIGIT, CD27 and ICOS between P1 and P2 subgroups.

As summarized in Figure 5A, P2 subgroup had significantly higher number of tumor-infiltrating CD8^+^ T cells, CD4^+^ T cells, helper T cells, activated natural killer cells and lower number of M2 macrophage than P1, suggesting an enhanced immunosurveillance in P2 subgroup. Of note, the regulatory T cell, a well-known member of suppressor T cell population, displayed a significantly higher fraction in P2 than in P1 subgroup.

As for antigen presenting genes, we found that P2 had dramatically higher expressions of HLA-A, HLA-B, HLA-C, TAP1 and B2M than P1 (Figure 5B), which are main regulatory genes for human MHC class I cell surface receptors and thus activate cytotoxic T cells. Besides, P2 subgroup was also associated with higher expressions of GZMA and RPRF (Figure 5C), two important regulatory genes for cytolytic activity. These findings partially accounted for the above results that tumors in P2 subgroup had stronger immunogenicity and therefore presented higher numbers of active immune cell infiltrations.

However, in terms of immunomodulator genes, P2 subgroup was significantly associated with higher expressions of PD-1, PD-L1, PD-L2, LAG3, TIM3, CTLA-4, CCR4 and TIGIT than P1 subgroup (Figure 5D), all of which are key genes of T-cell exhaustion markers. Besides, the expressions of CD27 and ICOS were also significantly higher in P2 than P1 subgroup. Therefore, it indicated that prognostic pseudogenes played a critical role in host antitumor response and might serve as potential targets for immunotherapy.

### Pseudogene-miRNA-target gene regulatory networks

**Figure 6:**
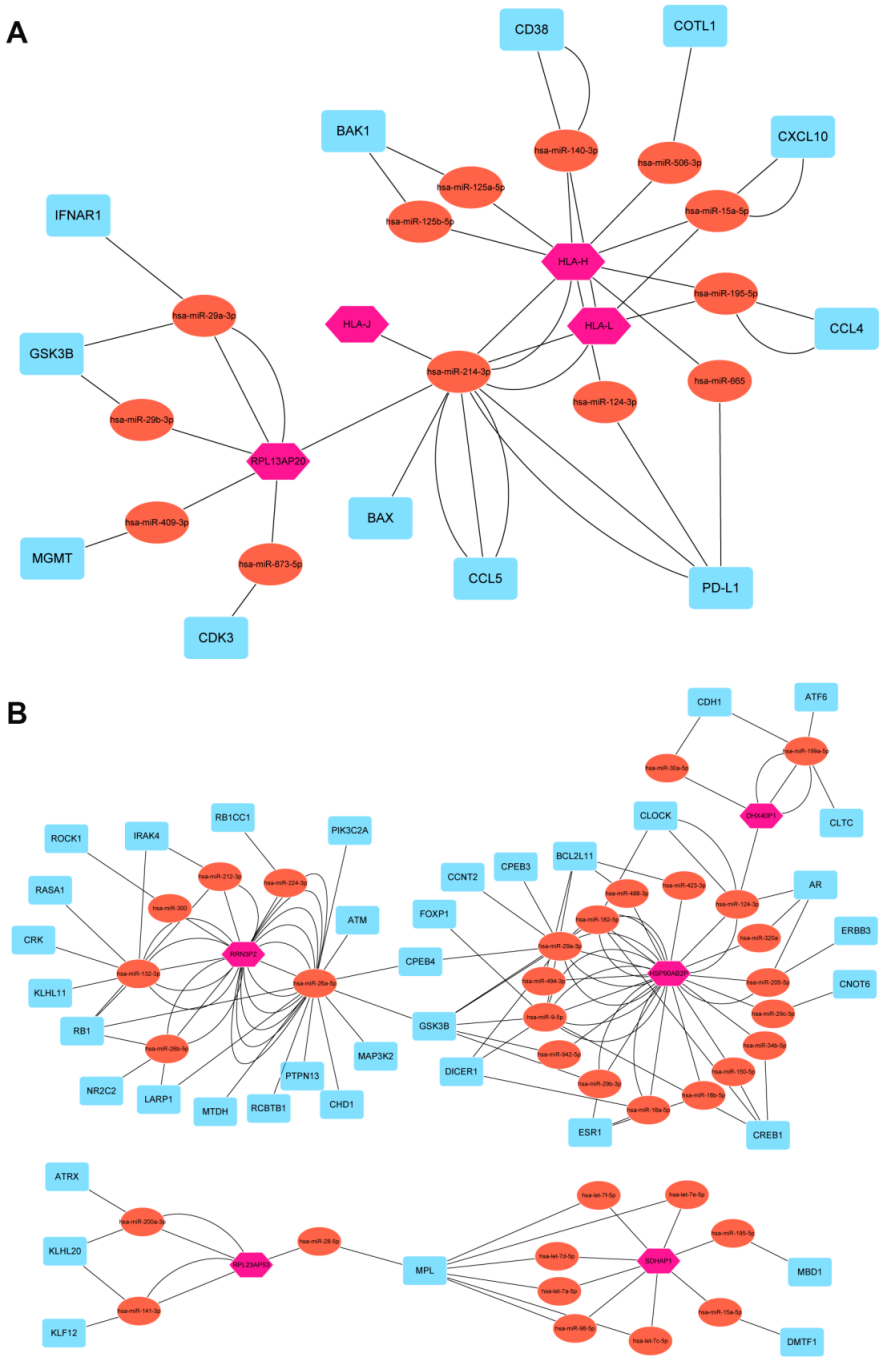
**Pseudogene-miRNA-target gene regulatory networks.** Nine pseudogenes together with binding miRNAs and target genes with |r| ≥ 0.3 and P < 0.05 were used to construct the pseudogene-miRNA-target gene regulatory networks by subgroups of tumor-suppressor pseudogenes (**A**) and oncogene pseudogenes (**B**). Pink hexagons represented pseudogenes, which are located at the cores of the networks. Tomato ellipses and blue round rectangles stand for binding miRNAs and target genes, respectively.

To elucidate the underlying mechanism how pseudogenes regulated anti-tumor immune response, we built a pseudogene-miRNA-target gene regulatory network. Potential miRNAs binding to the 15 pseudogenes were identified using the dreamBase database (Supplementary Table 4). Pearson correlation analysis was used to calculate expression correlations between each pseudogene and its miRNA target genes. Target genes with | r | **≥** 0.3 and P < 0.05 were picked up (Supplementary Table 5). Ultimately, 4 tumor-suppressor pseudogenes (HLA-J, HLA-H, HLA-L and RPL13AP20) together with 13 microRNAs and 19 targeted genes, and 5 oncogene pseudogenes (HSP90AB2P, DHX40P1, RRN3P2, SDHAP1 and RPL23AP53) together with 35 microRNAs and 43 targeted genes, were used to construct the pseudogene-miRNA-target gene regulatory networks and visualized using Cytoscape (Figure 6). As results indicated, pseudogene HLA-L upregulated the expression of PD-L1 by competitively binding hsa-miR-124-3p, which explained the higher expression of PD-L1 in P2 subgroup. Pseudogene HLA-H, acting as decoy of has-miR-140-3p, upregulated the expression of CD38 and then upregulated the infiltrations of many immune cells (including CD4^+^ T, CD8^+^ T, B lymphocytes and natural killer cells) by signal transduction and calcium signaling (Figure 6A). Other pseudogenes played regulatory roles in signaling pathways involving cell proliferation, oncogenic transformation, cell survival, cell migration, and intracellular protein trafficking as ceRNAs (Figure 6B). The pseudogene-miRNA-target gene regulatory networks partially clarified the mechanism how pseudogenes participated in regulating the antitumor immune response in breast cancer.

## DISCUSSION

In the current study, 15 pseudogenes were identified as promising prognostic indicators for breast cancer by univariate Cox regression analysis and classified into tumor-suppressor pseudogenes (NCF1C, HLA-DRB6, HLA-DPB2, HLA-J, HLA-H, HLA-L, RPL13AP20) and oncogene pseudogenes (PGM5P2, HERC2P4, HSP90AB2P, DHX40P1, RRN3P3, RRN3P2, SDHAP1, RPL23AP53) based on their different effects in clinical outcomes using TCGA dataset. Then a risk score model was constructed based on the 15 prognostic pseudogenes, and was found good in predicting prognosis in breast cancer. The prognostic value for these 15 pseudogenes and the risk score signature was further validated in EGA dataset. What’s more, we also found that the expression pattern of these 15 prognostic pseudogenes was significantly associated with antitumor immune response in terms of tumor-infiltrating immune cell compositions, antigen presenting genes expression, immunomodulator genes expression and cytolytic activity. Pseudogene-miRNA-target gene regulatory networks were further performed to elucidate the underlying mechanisms. To the best of our knowledge, this is the first study to systemically clarify the prognostic value of pseudogenes in breast cancer, and their regulatory roles in host antitumor immune response.

Pseudogenes, belonging to the non-coding RNA family, are pervasively transcribed in the genome [23]. The noncoding transcripts range from 100 bp to approximately 100 kilobases (kb) in length and lack significant open reading frames, which once mislead people to consider pseudogenes as “genetic fossils”. However, recent evidence suggests that pseudogenes can play important regulatory functions in diverse human diseases [24]. They were found to contain miRNA-binding elements and thus increase their parental transcripts by acting as competitive endogenous RNAs (ceRNA) [25, 26]. This significant finding worked as a strong cornerstone for studying the biological roles of pseudogenes in cancer.

Although currently there are no published studies concerning the prognostic effects of pseudogenes in breast cancer, previous studies have indicated the crucial roles of pseudogenes in tumorigenesis, tumor development and progression of other malignant tumors. For instant, pseudogene PTENP1 could suppress the progression of clear-cell renal cell carcinoma by functioning as a ceRNA [27]. Pseudogenes PKMP3, AC027612.4, HILS1, RP5-1132H15.3 and HSPB1P1 were identified as prognostic predictors for lower-grade gliomas [28]. In addition, pseudogenes ANXA2P2, EEF1A1P9, FER1L4, HILS1, and RAET1K were found to be significantly correlated with glioma survival [29]. What’ more, pseudogene RNA5SP141 was able to strongly enhance the RIG-I-mediated antiviral immunity response to herpes simplex virus 1 [30]. In the current study, we identified 15 prognostic pseudogenes that significantly associated with clinical outcomes in breast cancer. They were further classified into two functional subgroups, tumor-suppressor pseudogenes (NCF1C, HLA-DRB6, HLA-DPB2, HLA-J, HLA-H, HLA-L, RPL13AP20) and oncogene pseudogenes (PGM5P2, HERC2P4, HSP90AB2P, DHX40P1, RRN3P3, RRN3P2, SDHAP1, RPL23AP53) based on their different effects in clinical outcomes. Then we constructed a risk score model based on the 15 prognostic pseudogenes by LASSO Cox regression, which was found good in predicting prognosis in breast cancer. All in all, our study provides promising prognostic predictors for breast cancer patients, which can better execute the principle of precise medicine.

To the best of our knowledge, this is the first study concerning the correlation between pseudogenes and host antitumor immune response in breast cancer. Surprisingly, our study revealed that the expression pattern of the 15 prognostic pseudogenes was significantly associated with active tumor-infiltrating CD8^+^ T cells, CD4^+^ T cells, helper T cells and activated natural killer cells, as well as the expressions of HLA-A, HLA-B, HLA-C, TAP1, B2M, GZMA and RPRF. What’s more, T cell exhausted markers including PD-1, PD-L1, PD-L2, LAG3, TIM3, CTLA-4, CCR4 and TIGIT were also significantly associated with the expression pattern of pseudogenes. In addition, pseudogene-miRNA-target gene regulatory networks were further performed to elucidate the underlying mechanisms and demonstrated 4 tumor-suppressor pseudogenes (HLA-J, HLA-H, HLA-L and RPL13AP20) together with 13 microRNAs and 19 targeted genes, and 5 oncogene pseudogenes (HSP90AB2P, DHX40P1, RRN3P2, SDHAP1 and RPL23AP53) together with 35 microRNAs and 43 targeted genes as main regulatory factors. This large network could provide robust evidence for the further study about the biological roles of pseudogenes in host antitumor immune response in breast cancer.

One limitation of this study needs to be taken into consideration. All the analyses in the current study were based on the bioinformatics tools, therefore, further experimental validation is warranted to confirm the results of our study.

To sum up, we identified 15 prognostic pseudogenes and demonstrated that their expression pattern was significantly correlated with the clinicopathological features, survival outcomes and expressions of immunomodulator genes in breast cancer. This current study provided comprehensive evidence for further study of pseudogenes in breast cancer, and shed new light on the epigenetic regulation of antitumor immune response.

## MATERIALS AND METHODS

### Data sources

Genome and transcript sequences and annotation were obtained from the human genome (GRCh37), version 19 (Ensembl 74) (https://www.encodeproject.org/). A list of pseudogenes was collected from Vega databases (http://vega.archive.ensembl.org/index.html) and psiCube databases according to online pseudogene posted data (http://pseudogene.org/) [31, 32]. The breast cancer gene expression data and corresponding clinical information were obtained from TCGA data portal (http://firebrowse.org/) and EGA dataset (https://ega-archive.org/) by access number (EGAS00001001908). Altogether, 775 samples from TCGA and 50 samples from EGA with pseudogene expression data and corresponding clinical data were included. Immune cell fraction data were downloaded through CIBERSORT (https://cibersort.stanford.edu/) [33, 34]. The antigen presenting genes and immunomodulator genes were obtained from TCIA (https://tcia.at/home) [35]. miRNAs binding to pseudogenes were extracted from the dreamBase database (http://rna.sysu.edu.cn/dreamBase/index.php) [36]. miRNA target genes were identified using the miRTarBase (http://mirtarbase.mbc.nctu.edu.tw/php/index.php) [37].

### Screening for prognostic pseudogenes by cox proportional hazard regression analysis

Since most of the pseudogenes were not expressed, we first excluded those with the expression values (RPKM) less than 1. Then, univariate Cox proportional hazard regression was performed to screen for the candidate pseudogenes closely associated with overall survival. After these two steps, 15 pseudogenes were identified significantly associated with survival outcomes in TCGA dataset (P < 0.05) and further validated in EGA dataset.

### Construction of the risk score model

Based on LASSO Cox regression algorithm [38], a L1-penalized regression on the strength of the highest lambda value selected by means of a 1,000 cross-validations (‘1-se’ lambda) was conducted to further identify the regression coefficients of the 15 prognostic pseudogenes. Then a survival risk score model was established by the LASSO coefficients (β) as follows:

$$\text{Risk}\;\text{score}=\sum_{i=1}^{n}Geneexpri\times \beta i.$$

The breast cancer patients were divided into low or high-risk groups based on the median risk score. The receiver operating characteristic (ROC) curve and area under the curve (AUC) were conducted to estimate the prediction accuracy of the risk score model. Each prognostic pseudogene was dichotomized into low or high expression level, with cut-off value defining as the median expression value. Kaplan-Meier plots and Log-rank test were utilized to evaluate and compare the survival rate between subgroups. All the analyses mentioned above were performed using TCGA data as the training set and EGA data as the validation set. Univariate and multivariate Cox regression analyses were carried out to determine the prognostic value of the risk score and various clinical characteristics.

### Consensus clustering analysis

To investigate the functional roles of pseudogenes in breast cancer, we clustered the patients into different subgroups by the R package “ConsensusClusterPlus” (50 iterations, resample rate of 80%, and Pearson correlation) based on the expression levels of the prognostic pseudogenes in TCGA dataset [39].

### Functional analysis of the prognostic pseudogenes

To better understand the association between prognostic pseudogenes and malignancy of breast cancer, GO pathway analysis, KEGG analysis and GSEA [40] were carried out to functionally annotate genes that differentially expressed in different subgroups by using the R package “clusterProfiler” [41].

### Immune cell infiltration, immune response and immune cytolysis

CIBERSORT [33], a bioinformatic deconvolution algorithm to calculate immune cell composition from their gene expression profiles, was used to assess tumor-infiltrating cell compositions in diverse tumors [42]. The immune cell fractions, expressions of antigen presenting genes, immunomodulator genes [43] and immune cytolysis genes [44] were compared in different subgroups by Wilcoxon signed-rank test.

### Pseudogene-miRNA-target gene regulatory networks

miRNAs binding to prognostic pseudogenes were obtained from the dreamBase database [36]. miRNA target genes with at least one strong experimental method (reporter assay or western blot) were extracted by the miRTarBase [37]. Pearson analysis was conducted to calculate expression correlation between pseudogenes and miRNA target genes. Target genes conforming to | r | **≥** 0.3 and P < 0.05 were selected and applied to construct pseudogene-miRNA-target gene regulatory networks using Cytoscape 3.7.1.

### Statistical analysis

One-way ANOVA and t test were carried out to compare the expression levels of prognostic pseudogenes in different subgroups differentiated by lymph node status, molecular subtypes and distant metastasis status. Chi-square test was used to evaluate the differences of clinicopathological characteristics between subgroups identified by consensus clustering of pseudogenes. All statistical analyses were performed using R software (http://www.r-project.org/) and Bioconductor (http://bioconductor.org/). A two-sided p value < 0.05 was considered statistically significant in all analyses.

## Supplementary Material

Supplementary Figures

Supplementary Tables
