# Supplementary material for: Identification of a 15-pseudogene based prognostic signature for predicting survival and antitumor immune response in breast cancer
Source: Aging (Albany NY). 2020 Dec 16;13(10):14499–521. doi: 10.18632/aging.103735 (PMC8202842; doi:10.18632/aging.103735)
Supplement: Supplementary Figures [file aging-13-103735-s001.pdf]

SUPPLEMENTARY FIGURES

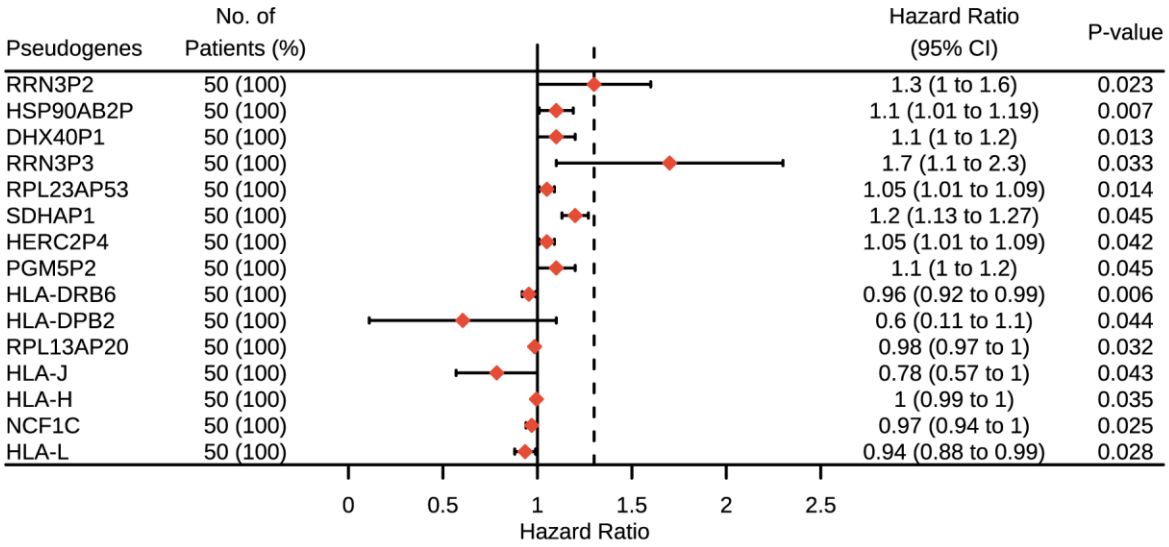

Supplementary Figure 1. Validation of the 15 prognostic pseudogenes in EGA dataset. The hazard ratios (HR), 95% confidence intervals (CI) calculated by univariate Cox proportional hazard regression of the 15 prognostic pseudogenes in EGA dataset.

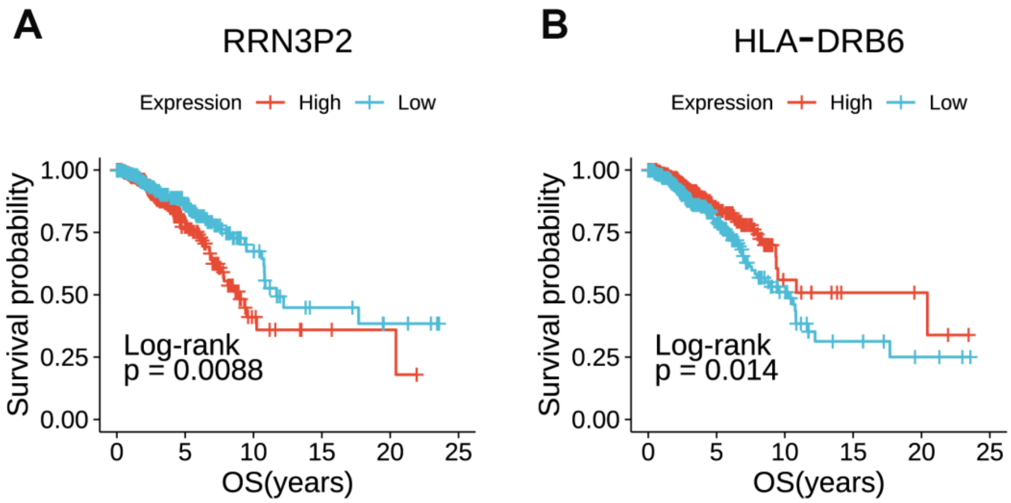

Supplementary Figure 2. Correlation between the expression levels of RRN3P2 (**A**) and HLA-DRB6 (**B**) and overall survival in patients with breast cancer from TCGA dataset.

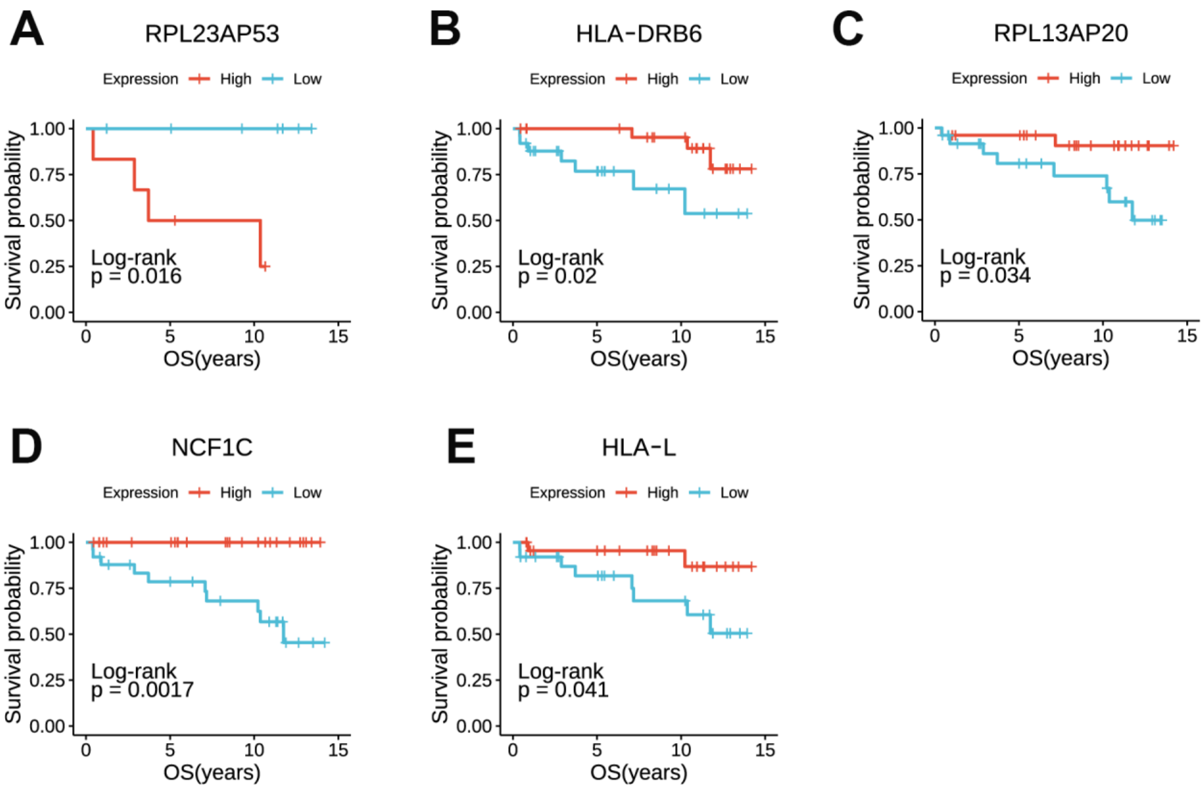

Supplementary Figure 3. Correlation between the expression levels of RPL23AP53 (**A**), HLA-DRB6 (**B**), RPL13AP20 (**C**), NCF1C (**D**) and HLA-L (**E**) and overall survival in patients with breast cancer from EGA dataset.
