# Supplementary material for: Identification of a 15-pseudogene based prognostic signature for predicting survival and antitumor immune response in breast cancer
Source: Aging (Albany NY). 2020 Dec 16;13(10):14499–521. doi: 10.18632/aging.103735 (PMC8202842; doi:10.18632/aging.103735)
Supplement: Supplementary Tables [file aging-13-103735-s002.pdf]

SUPPLEMENTARY TABLES

Supplementary Table 1. The coefficients of the 15 prognostic pseudogenes by LASSO.

Pseudogenes	Coefficients
NCF1C	0.000000000
HLA-DRB6	-0.002846803
HLA-DRB2	-0.104956422
HLA-J	-0.057654172
HLA-H	0.000000000
HLA-L	-0.013823392
RPL13AP20	-0.001763734
PGM5P2	0.000000000
HERC2P4	0.000000000
HSP90AB2P	0.000000000
DHX40P1	0.044932918
RRN3P3	0.000000000
RRN3P2	0.226104532
SDHAP1	0.046539494
RPL23AP53	0.021710806

Supplementary Table 2. Clinicopathological features stratified by high-risk and low-risk subgroups.

Variable	High-risk, n (%)	Low-risk, n (%)	*P*
No. of Patients	387(49.94)	388(50.06)	-
Age at diagnosis, years			0.603
≤ 50	116(14.97)	124(16.00)	
> 50	271(34.96)	264(34.06)	
ER status			8e-08
Negative	53(6.84)	118(15.23)	
Positive	311(40.13)	257(33.16)	
Unknown	23(2.97)	15(1.68)	
PR status			6e-04
Negative	97(12.52)	145(18.71)	
Positive	265(34.19)	228(29.42)	
Unknown	25(3.22)	15(1.94)	
HER-2 status			0.904
Negative	269(34.71)	272(35.10)	
Positive	65(8.39)	67(8.65)	
Unknown	53(6.84)	49(6.32)	
Molecular subtypes			4e-06
Normal-like	42(5.42)	89(11.48)	
Luminal A	26(3.35)	39(5.03)	
Luminal B	206(26.58)	166(21.42)	
HER2 positive	102(13.16)	75(9.68)	
Basal-like	6(0.77)	16(2.06)	
Unknown	5(0.64)	3(0.39)	
T stage			0.688
T1	105(13.55)	100(12.90)	
T2	223(28.77)	235(30.32)	
T3	41(5.29)	42(5.42)	
T4	17(2.19)	10(1.29)	
Unknown	1(0.13)	1(0.13)	
Lymph node stage			0.037
N0	168(21.68)	193(24.90)	
N1	137(17.68)	127(16.39)	
N2	52(6.71)	40(5.16)	
N3	18(2.32)	25(3.23)	
Unknown	12(1.55)	3(0.39)	
Distance metastasis status			0.248
Negative	347(44.77)	354(45.68)	
Positive	6(0.77)	10(1.29)	
Unknown	34(4.39)	24(3.10)	
Vital status			0.070
Alive	318(41.03)	338(43.61)	
Dead	69(8.90)	50(6.45)	

Abbreviations: ER: estrogen receptor; PR: progesterone receptor; HER-2: human epidermal growth receptor-2.
* Evaluated by Chi-square test.

Supplementary Table 3. Clinicopathological features stratified by P1 and P2 subgroups.

Variable	P1, n (%)	P2, n (%)	*P*
No. of Patients	362(46.71)	413(53.29)	-
Age at diagnosis, years			0.709
≤ 50	115(14.84)	125(16.12)	
> 50	247(31.87)	288(37.16)	
ER status			0.028
Negative	66(8.52)	105(13.55)	
Positive	275(35.48)	293(37.81)	
Unknown	21(2.71)	15(1.94)	
PR status			0.413
Negative	107(13.81)	135(17.42)	
Positive	233(30.06)	260(33.55)	
Unknown	22(2.84)	18(2.32)	
HER-2 status			0.358
Negative	250(32.25)	291(37.55)	
Positive	58(7.48)	74(9.55)	
Unknown	54(6.97)	48(6.19)	
Molecular subtypes			0.002
Normal-like	47(6.06)	84(10.84)	
Luminal A	26(3.35)	39(5.03)	
Luminal B	180(23.23)	192(24.77)	
HER2 positive	95(12.25)	82(10.58)	
Basal-like	7(0.90)	15(1.94)	
Unknown	7(0.90)	1(0.13)	
T stage			0.075
T1	93(12.00)	112(14.45)	
T2	211(27.23)	247(31.87)	
T3	37(4.77)	46(5.94)	
T4	20(2.58)	7(0.90)	
Unknown	1(0.13)	1(0.13)	
Lymph node stage			0.047
N0	153(19.74)	208(26.84)	
N1	132(17.03)	132(17.03)	
N2	48(6.19)	44(5.68)	
N3	18(2.32)	25(3.23)	
Unknown	11(1.42)	4(0.52)	
Distance metastasis status			0.441
Negative	325(41.94)	376(48.52)	
Positive	6(0.77)	10(1.29)	
Unknown	31(4.00)	27(3.48)	
Vital status			0.560
Alive	303(39.1)	353(45.5)	
Dead	59(7.61)	60(7.74)	

Abbreviations: P1: patient subgroup 1; P2: patient subgroup 2; ER: estrogen receptor; PR: progesterone receptor; HER-2: human epidermal growth receptor-2.
* Evaluated by Chi-square test.

Supplementary Table 4. Potential miRNAs binding to the 15 prognostic pseudogenes identified by dreamBase.

Pseudogene	miRNA
**NCF1C**	0
**HLA-DRB6**	0
**HLA-DRB2**	0
**HLA-J**	hsa-miR-1193; hsa-miR-140-3p; hsa-miR-15a-5p; hsa-miR-15b-5p; hsa-miR-16-5p; hsa-miR-195-5p; hsa-miR-214-3p; hsa-miR-2278; hsa-miR-3619-5p; hsa-miR-3918; hsa-miR-424-5p; hsa-miR-4428; hsa-miR-4726-5p; hsa-miR-497-5p; hsa-miR-589-5p; hsa-miR-6838-5p; hsa-miR-761
**HLA-H**	hsa-miR-124-3p; hsa-miR-125a-5p; hsa-miR-125b-5p; hsa-miR-1343-3p; hsa-miR-140-3p; hsa-miR-143-3p; hsa-miR-15a-5p; hsa-miR-15b-5p; hsa-miR-16-5p; hsa-miR-195-5p; hsa-miR-214-3p; hsa-miR-22-3p; hsa-miR-2278; hsa-miR-296-5p; hsa-miR-3127-5p; hsa-miR-3184-5p; hsa-miR-3200-5p; hsa-miR-323a-3p; hsa-miR-323b-3p; hsa-miR-3605-3p; hsa-miR-3619-5p; hsa-miR-362-5p; hsa-miR-380-3p; hsa-miR-3918; hsa-miR-423-5p; hsa-miR-424-5p; hsa-miR-4319; hsa-miR-4640-5p; hsa-miR-4726-5p; hsa-miR-4770; hsa-miR-497-5p; hsa-miR-500b-5p; hsa-miR-506-3p; hsa-miR-514a-5p; hsa-miR-532-3p; hsa-miR-605-3p; hsa-miR-6088; hsa-miR-665; hsa-miR-6746-3p; hsa-miR-6783-3p; hsa-miR-6838-5p; hsa-miR-744-5p; hsa-miR-761; hsa-miR-766-5p
**HLA-L**	hsa-miR-140-3p; hsa-miR-15a-5p; hsa-miR-15b-5p; hsa-miR-16-5p; hsa-miR-195-5p; hsa-miR-214-3p; hsa-miR-2278; hsa-miR-296-5p; hsa-miR-3127-5p; hsa-miR-335-5p; hsa-miR-3619-5p; hsa-miR-370-3p; hsa-miR-380-3p; hsa-miR-3918; hsa-miR-424-5p; hsa-miR-4428; hsa-miR-4726-5p; hsa-miR-497-5p; hsa-miR-500b-5p; hsa-miR-6838-5p; hsa-miR-6893-3p; hsa-miR-761
**RPL13AP20**	hsa-miR-1224-5p; hsa-miR-193a-5p; hsa-miR-214-3p; hsa-miR-296-3p; hsa-miR-29a-3p; hsa-miR-29b-3p; hsa-miR-29c-3p; hsa-miR-3619-5p; hsa-miR-3681-5p; hsa-miR-409-3p; hsa-miR-452-5p; hsa-miR-4664-3p; hsa-miR-4676-3p; hsa-miR-486-5p; hsa-miR-526b-5p; hsa-miR-532-5p; hsa-miR-6512-3p; hsa-miR-665; hsa-miR-6849-5p; hsa-miR-761; hsa-miR-766-5p; hsa-miR-873-5p; hsa-miR-892c-3p
**PGM5P2**	hsa-miR-328-3p
**HERC2P4**	hsa-miR-146a-5p; hsa-miR-146b-5p; hsa-miR-181a-5p; hsa-miR-181b-5p; hsa-miR-181c-5p; hsa-miR-181d-5p; hsa-miR-205-5p; hsa-miR-4262; hsa-miR-7153-5p
**HSP90AB2P**	hsa-miR-124-3p; hsa-miR-1252-5p; hsa-miR-128-3p; hsa-miR-142-5p; hsa-miR-144-5p; hsa-miR-150-5p; hsa-miR-182-5p; hsa-miR-18a-5p; hsa-miR-18b-5p; hsa-miR-205-5p; hsa-miR-2115-3p; hsa-miR-216a-3p; hsa-miR-2682-5p; hsa-miR-29a-3p; hsa-miR-29b-3p; hsa-miR-29c-3p; hsa-miR-3187-3p; hsa-miR-320a; hsa-miR-320b; hsa-miR-320c; hsa-miR-320d; hsa-miR-345-3p; hsa-miR-34b-5p; hsa-miR-365a-3p; hsa-miR-365b-3p; hsa-miR-3681-3p; hsa-miR-376a-3p; hsa-miR-376b-3p; hsa-miR-376c-3p; hsa-miR-380-3p; hsa-miR-423-3p; hsa-miR-4429; hsa-miR-449c-5p; hsa-miR-4735-3p; hsa-miR-4761-5p; hsa-miR-4766-3p; hsa-miR-4766-5p; hsa-miR-488-3p; hsa-miR-494-3p; hsa-miR-496; hsa-miR-506-3p; hsa-miR-514a-5p; hsa-miR-515-5p; hsa-miR-519e-5p; hsa-miR-5590-3p; hsa-miR-577; hsa-miR-616-3p; hsa-miR-670-3p; hsa-miR-670-5p; hsa-miR-766-5p; hsa-miR-874-3p; hsa-miR-9-5p; hsa-miR-942-5p; hsa-miR-944
**DHX40P1**	hsa-miR-124-3p; hsa-miR-1271-5p; hsa-miR-1306-5p; hsa-miR-186-5p; hsa-miR-199a-5p; hsa-miR-199b-5p; hsa-miR-22-3p; hsa-miR-30a-5p; hsa-miR-30b-5p; hsa-miR-30c-5p; hsa-miR-30d-5p; hsa-miR-30e-5p; hsa-miR-33a-5p; hsa-miR-33b-5p; hsa-miR-3612; hsa-miR-506-3p; hsa-miR-545-5p; hsa-miR-625-3p; hsa-miR-628-5p; hsa-miR-650; hsa-miR-7151-5p; hsa-miR-96-5p
**RRN3P3**	0
**RRN3P2**	hsa-miR-1297; hsa-miR-132-3p; hsa-miR-191-5p; hsa-miR-212-3p; hsa-miR-224-3p; hsa-miR-26a-5p; hsa-miR-26b-5p; hsa-miR-300; hsa-miR-381-3p; hsa-miR-4465;

	hsa-miR-4524a-5p; hsa-miR-4524b-5p; hsa-miR-522-3p; hsa-miR-532-5p
**SDHAP1**	hsa-let-7a-5p; hsa-let-7b-5p; hsa-let-7c-5p; hsa-let-7d-5p; hsa-let-7e-5p; hsa-let-7f-5p; hsa-let-7g-5p; hsa-let-7i-5p; hsa-miR-105-5p; hsa-miR-1249-3p; hsa-miR-1301-3p; hsa-miR-136-5p; hsa-miR-15a-5p; hsa-miR-15b-5p; hsa-miR-16-5p; hsa-miR-195-5p; hsa-miR-216a-5p; hsa-miR-2355-5p; hsa-miR-2681-3p; hsa-miR-3150a-3p; hsa-miR-3529-5p; hsa-miR-361-3p; hsa-miR-3622b-5p; hsa-miR-379-5p; hsa-miR-424-5p; hsa-miR-4458; hsa-miR-4500; hsa-miR-4731-5p; hsa-miR-4761-3p; hsa-miR-485-5p; hsa-miR-491-5p; hsa-miR-495-3p; hsa-miR-497-5p; hsa-miR-5047; hsa-miR-505-3p; hsa-miR-516b-5p; hsa-miR-542-3p; hsa-miR-543; hsa-miR-545-3p; hsa-miR-5688; hsa-miR-5691; hsa-miR-6763-5p; hsa-miR-6805-3p; hsa-miR-6838-5p; hsa-miR-6884-5p; hsa-miR-7853-5p; hsa-miR-98-5p
**RPL23AP53**	hsa-miR-1343-3p; hsa-miR-141-3p; hsa-miR-200a-3p; hsa-miR-214-5p; hsa-miR-28-5p; hsa-miR-3139; hsa-miR-376a-3p; hsa-miR-376b-3p; hsa-miR-6783-3p; hsa-miR-708-5p

Supplementary Table 5. miRNA targeted genes correlated with their pseudogenes at | r | ≥ 0.3 and P < 0.05.

Pseudogene	miRNA targeted genes
**NCF1C**	0
**HLA-DRB6**	0
**HLA-DRB2**	0
**HLA-J**	CCL5
**HLA-H**	CD274; BAK1; CD38; CXCL10; CCL4; CCL5; COTL1
**HLA-L**	CD38; CXCL10; CCL4; CCL5; CD274
**RPL13AP20**	BAX; GSK3B; IFNAR1; GSK3B; MGMT; CDK3
**PGM5P2**	0
**HERC2P4**	0
**HSP90AB2P**	AR; BCL2L11; CCNT2; CLOCK; CNOT6; CPEB3; CPEB4; CREB1; DICER1; ERBB3; ESR1; FOXP1; GSK3B
**DHX40P1**	CLOCK; ATF6; CDH1; CLTC
**RRN3P3**	0
**RRN3P2**	ATM; CHD1; CPEB4; CRK; GSK3B; IRAK4; KLHL11; LARP1; MAP3K2; MTDH; NR2C2; PIK3C2A; PTPN13; RASA1; RB1; RB1CC1; RCBTB1; ROCK1
**SDHAP1**	MPL; DMTF1; MBD1; MPL
**RPL23AP53**	KLF12; KLHL20; ATRX; MPL
